# LOCAS: multilabel mRNA localization with supervised contrastive learning

**DOI:** 10.1093/bib/bbaf441

**Published:** 2025-08-27

**Authors:** Abrar Rahman Abir, Md Toki Tahmid, M Saifur Rahman

**Affiliations:** Department of Computer Science and Engineering, Bangladesh University of Engineering and Technology, Dhaka-1000, Bangladesh; Department of Computer Science and Engineering, Bangladesh University of Engineering and Technology, Dhaka-1000, Bangladesh; Department of Computer Science and Engineering, Bangladesh University of Engineering and Technology, Dhaka-1000, Bangladesh

**Keywords:** mRNA subcellular localization, contrastive learning, RNA language model

## Abstract

The subcellular localization of messenger RNAs (mRNAs) plays a crucial role in gene regulation, ensuring precise spatial and temporal control of protein synthesis. Traditional computational approaches for mRNA localization have primarily relied on single-label classification models, which fail to capture the inherent multi-label nature of mRNA localization. Recent advancements have introduced deep learning-based multi-label prediction frameworks; however, existing methods often lack an effective way to model the relationships between multiple localizations. In this paper, we propose Localization with Supervised Contrastive Learning (LOCAS), a novel approach for multi-label mRNA subcellular localization prediction. LOCAS integrates an RNA language model (RiNALMo) to generate high-quality sequence embeddings and employs supervised contrastive learning (SCL) to refine the embedding space, ensuring biologically meaningful clustering of RNA sequences. To handle overlapping labels, we introduce an overlap-threshold-based similarity measure during contrastive training. Finally, we leverage an ML-Decoder, which utilizes a cross-attention mechanism to enhance multi-label classification performance. We evaluate LOCAS on two benchmark datasets, RNALocate and RNALocate V2.0, demonstrating state-of-the-art performance across all evaluation metrics. Extensive ablation studies validate the effectiveness of our approach, highlighting the contributions of contrastive learning and ML-decoder in improving multi-label classification. Our results suggest that integrating RNA sequence representation learning with SCL offers a powerful and scalable solution for mRNA localization prediction.

## Introduction

The subcellular localization of messenger RNAs (mRNAs) is a critical process in the regulation of gene expression that ensures the spatial and temporal control necessary for proper cellular function. The phenomenon of mRNA localization, first identified in the early 1980s, has since been recognized as a prevalent mechanism across various cell types and organisms. For example, studies have shown that actin mRNA is localized within the cytoplasm during the development of ascidian embryos, an early discovery that underscored the importance of mRNA distribution within cells [1].

mRNAs are not distributed uniformly within the cell but are instead localized to specific compartments, a process essential for various cellular functions such as establishing cell polarity, motility, and guiding embryonic development. This localization allows for the precise control of protein synthesis, which is particularly important in complex cells. For example, localized mRNA translation is crucial in neurons, where it plays a role in synaptic plasticity and memory formation [2, 3]. The transport and localization of mRNAs are directed by cis-regulatory elements, often referred to as zipcodes, which are recognized by RNA-binding proteins. These interactions facilitate the formation of ribonucleoprotein complexes, which are then transported along the cytoskeleton to specific locations within the cell [4, 5].

The asymmetric distribution of mRNAs has been shown to provide several advantages, including low transport costs and the prevention of ectopic protein activity during translocation. Additionally, this process allows for rapid local responses to external stimuli, which is critical in processes such as synaptic signaling in neurons [5–7]. The precise localization of mRNAs also plays a role in protecting mRNAs from degradation, further ensuring that they are available for translation at the correct time and place within the cell [5, 6].

Beyond its role in normal cellular functions, mRNA localization is also critical in disease. Aberrant mRNA localization has been implicated in a variety of human diseases, including neurodevelopmental disorders such as fragile X syndrome, embryonal disorders, Alzheimer’s disease, and cancer [5, 8–11]. These associations highlight the importance of understanding the mechanisms underlying mRNA localization, as they could provide insights into disease pathology and potential therapeutic targets.

The traditional methods used to study mRNA localization, such as *in situ* hybridization (ISH) and high-throughput RNA sequencing, have been invaluable in advancing our understanding of this process [3]. However, these methods are often time-consuming and costly, limiting their use in large-scale studies. The need for more efficient and scalable methods has led to the development of computational approaches that can predict mRNA localization based on sequence data and other features. These computational tools offer a powerful complement to experimental techniques, allowing for the rapid and cost-effective analysis of mRNA localization across different cell types and conditions [5, 12–14].

Initial computational approaches to mRNA localization framed the problem as a single-label classification task, where each mRNA was predicted to localize to only one specific compartment. Among the early efforts in this domain, several methods stood out. RNATracker employed deep recurrent neural networks to predict mRNA localization based on raw sequence data and introduced a novel way to detect candidate zipcodes by masking parts of the sequence and evaluating their impact on prediction [15]. Another tool, iLoc-mRNA, utilized support vector machines (SVMs) to predict mRNA localization specifically in *Homo sapiens*, focusing on optimally preselected features to enhance prediction accuracy [16]. Similarly, mRNALoc was developed to predict the localization of mRNAs to five different subcellular compartments in eukaryotic cells, using SVMs with pseudo K-tuple nucleotide composition features [17]. SubLocEP further refined predictions by concentrating on specific cellular compartments while remaining within the single-label classification framework [18].

These single-label methods marked significant advancements in the early stages of computational mRNA localization prediction. However, they were inherently limited by the assumption that each mRNA localizes to only one compartment, which does not align with biological reality. Many mRNAs are known to localize in multiple compartments, fulfilling diverse roles within the cell [18, 19].

To address the limitations of single-label approaches, the field has shifted towards multi-label prediction models, which better capture the complexity of mRNA localization. Notable multi-label methods include DM3Loc, a deep learning-based model that utilized a multi-head self-attention mechanism to predict mRNA localization across multiple compartments simultaneously [5]. DM3Loc significantly improved the accuracy and biological relevance of mRNA localization predictions. Moreover, Clarion [3] employed an ensemble learning strategy based on XGBoost, enhancing the accuracy and robustness of multi-label mRNA localization predictions by considering label correlations and utilizing advanced feature selection methods. Furthermore, Allocator introduced the use of graph neural networks (GNNs) to incorporate RNA secondary structure information into the prediction model [20]. By integrating both sequence-based and structure-based features, Allocator provided a more comprehensive approach to predicting mRNA localization.

Multi-label data representation and prediction have recently been greatly studied in the field of computer vision [21–23] and natural language processing [24, 25]. MultiSupCon [26] proposed an approach to understand the embedding space of sets of images with supervised contrastive learning (SCL) with a projection embedding generated by an encoder. This helps the encoder to identify the distribution difference between different classes. In this paper, we have proposed a similar approach for RNA sequence representation learning. We generate the initial embedding space with a RNA language model (RiNALMo [27]). We perform clustering based on the RNA sequence embeddings with SCL and the clustering label is determined with an overlapping threshold on the multi-lables. Finally, we employ an ML-decoder [28] to effectively classify the fine-tuned embedding space (after SCL) into actual labels. With these components, we propose a multilabel RNA **Loca**lization with **S**upervised Contrastive Learning (LOCAS), where our main contributions are:

1. **RNA Language Model for Feature Representation:** We integrate an RNA language model (RiNALMo [27]) to generate an initial contextual embedding space for RNA sequences, enabling a more biologically meaningful representation of RNA sequences in the sub-cellular localization task. To the best of our knowledge, this is the first application of RNA language models in this domain.
2. **Supervised Contrastive Learning with Label Overlap Awareness:** Given the multi-label nature of the task, where RNAs may localize to multiple cellular compartments, we apply a SCL approach to identify distinct clusters of RNA sequences. Unlike traditional SCL methods that treat all classes equally, we introduce an overlap-aware similarity score based on an overlapping threshold to better capture inter-class relationships. This ensures that sequences sharing localization labels are grouped more effectively during training.
3. **Attention-Based ML-Decoder for Multi-Label Classification:** Instead of using a fixed MLP-based classification head, we adopt ML-Decoder, an attention-based prediction module designed for multi-label tasks. This component leverages cross-attention to dynamically learn label dependencies, enhancing the accuracy of multi-label classification.

Finally, we evaluate LOCAS on two widely used benchmark datasets, RNALocate and RNALocate V2.0. Through multiple ablation studies and hyperparameter tuning during SCL training, we determine the optimal parameters for training LOCAS. With this approach, we achieve state-of-the-art performance in RNA sub-cellular localization across all metrics compared to previously reported approaches.

## Methods

In this section, we first describe the datasets that are used for the RNA subcellular localization task. Then we dive into the architectural details of different components: feature representation 2.1.1, encoder network 2.1.2, SCL for RNA sequences 2.1.3, prediction head 2.1.4, and overall training pipeline 2.2.

### Dataset description

In this study we use two benchmark datasets: RNALocate [19] and RNALocate V2.0 [29].

**RNALocate** (February 2020) contains 17,870 mRNAs with experimentally verified subcellular localization in six compartments: nucleus, exosome, cytosol, ribosome, membrane, and endoplasmic reticulum (ER). Multiple localization labels were assigned when applicable.

**RNALocate V2.0** (June 2021) expands to 84,792 entries, covering 36,971 unique mRNAs with nine subcellular compartments: exosome, nucleus, cytosol, chromatin, nucleoplasm, ribosome, nucleolus, cytoplasm, and membrane.

As part of preprocessing, we removed duplicate entries and filtered out mRNAs with incomplete or inconsistent subcellular localization annotations. Sequences containing non-standard nucleotide characters were excluded to ensure data quality and sequences exceeding 6,000 nt were truncated by keeping the first and last 3,000 nt following [3]. To reduce redundancy, we applied a sequence similarity cutoff using CD-HIT [30], ensuring that no two sequences in the dataset exceed 80% identity, following [5]. These preprocessing steps were consistently applied to both RNALocate and RNALocate V2.0 to maintain dataset integrity.

Dataset details are provided in Table 1.

**Table 1:** Comparison of RNALocate and RNALocate V2.0 datasets

**Feature**	**RNALocate**	**RNALocate V2.0**
Release Date	Feb 2020	June 2021
Total Entries	17,870	84,792
Unique mRNAs	17,023	36,971
Subcellular Compartments	6	9
Compartments	Nucleus, Exosome, Cytosol, Ribosome, Membrane, ER	Exosome, Nucleus, Cytosol, Chromatin, Nucleoplasm, Ribosome, Nucleolus, Cytoplasm, Membrane
Sequence Length	200–30,000 nt	119–12,000 nt
Validation Method	Five-fold cross-validation	Independent test dataset with 3697 mRNAs.

#### Initial feature representation

Each RNA sequence in the dataset is first encoded with RNA language model RiNALMo [27]. RiNALMo is a character-level RNA language model that first splits all the nucleotides into different tokens. The vocabulary size is four: A, U, C, and G. For a given RNA sequence $X = \{N_{1}, N_{2}, N_{3}, \ldots , N_{L}\}$ where $L$ is the length of the RNA sequence, RiNALMo generates nucleotide-level features of dimension 1280. Thus, for a given input to the RiNALMo, the output from the language model encoder is a sequence of vectors $F = \{[CLS], f_{1}, f_{2}, f_{3}, \ldots , f_{N}\, [END]\}$ where each $f_{i} \in \mathbb{R}^{d}$, with $d = 1280$. To get a sequence level representation of the RNA, we consider the [CLS] token of the transformer’s output (which is the first token of the output). Thus, for a given RNA sequence $X$, RiNALMo generates a single feature vector of dimension $\mathbb{R}^{d}$, which is the initial feature representation for the sequence.

#### Encoder network

The initial feature representation vector $F$ is passed through an encoder network, $\text{Enc}(\cdot )$, that maps $x$ to a representation vector $r = \text{Enc}(F) \in \mathbb{R}^{D_{E}}$, where $D_{E} = 128$. In our implementation, the encoder combines convolutional feature extraction with self-attention mechanisms to effectively capture both local and global dependencies in sequential data. The architecture begins with a stack of three residual convolutional blocks, each progressively increasing the number of channels from 1 to 256. These residual convolutional blocks enhance feature extraction by allowing information to propagate through skip connections, preventing gradient vanishing and enabling deep feature learning. Each block consists of two convolutional layers, ReLU activation and batch normalization. After convolutional feature extraction, the encoder applies two stacked multi-head self-attention layers with 8 attention heads each. The attention layers refine the feature representations by capturing long-range dependencies across the sequence. To ensure stability and enhance learning, residual connections are incorporated around each attention block, followed by ReLU activation. Finally, the model applies adaptive average pooling to generate a fixed-dimensional representation, reducing the sequence into a compact feature vector. The output is passed through a fully connected layer to obtain the final encoded representation of the input. This architecture allows the encoder to effectively model both local contextual information via convolutions and global interactions via self-attention, making it well-suited for sequence-based learning tasks.


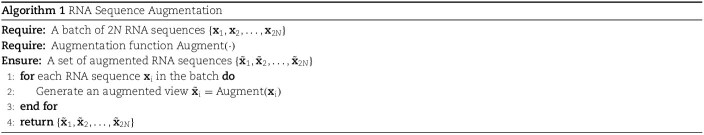


#### Supervised multilabel contrastive training

With the output from the encoder network $r = \text{Enc}(F)$, we pass this through a projection layer $ z= \text{Projection} (r) \in \mathbb{R}^{D_{P}}$, where $D_{P} = 64$. This projection layer is used for a contrastive training in the RNA dataset. We have multiple options for choosing the contrastive training scheme. Two primary methods that we can utilize are self-supervised contrastive training and supervised contrastive training. In the context of RNA data, we can leverage self-supervised contrastive learning to enhance the model’s ability to distinguish between different RNA sequences. For each RNA sequence, we can generate two distinct views by applying augmentations such as noise addition, random cropping, or jittering. These views are treated as positive pairs, while other RNA sequences in the batch are considered negative pairs. With these augmented views, we can train a self-supervised pre-training with Algorithm 2. For this algorithm to work properly, we have the requirement of two information for each sequence: the sequence’s contextually similar sequences and dissimilar sequences.

One major issue with this approach lies in the augmentation of the RNA dataset with Algorithm 1. Given the 3d structural information of the RNA molecules, we can apply augmentation techniques such as noise addition, random cropping, or jittering. However, we do not actually have any reliable way to perform this augmentation in sequence domain. RNA sequence data cannot be seamlessly transcribed into a different sequence containing similar information, as we can do in the natural language or vision domain [31].


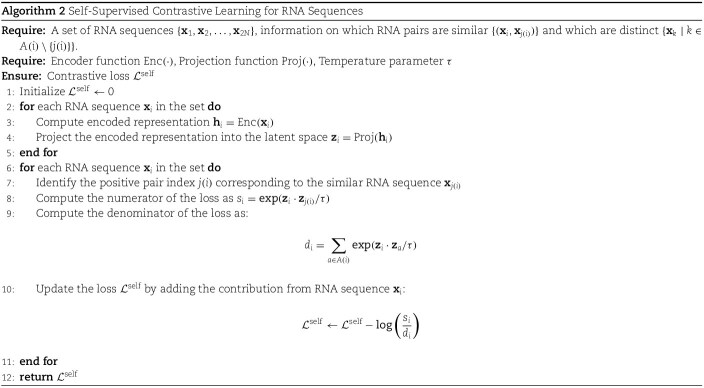


To tackle this issue, we can take advantage from the availability of the labels for each RNA sequence in the RNA subcellular localization dataset and use the concept of supervised contrastive loss as presented by [26]. In simple words, we consider the RNA sequences whose labels are the same as similar sequences during the contrastive pretraining and consider the other sequences as distant sequences. With Algorithm 3, we can utilize the label information to create the pair of similar and distinct sequences and leverage Algorithm 2 for contrastive training with this information.


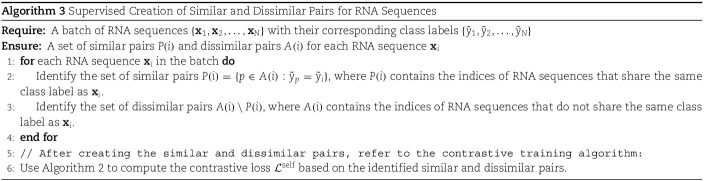


In multi-label problems, such as RNA subcellular localization, the supervised contrastive loss (as implemented in SupCon [32]) has a notable limitation. Specifically, it only treats two sets of labels as belonging to the same class when they are an exact match. However, in multi-label scenarios, it is rare to encounter identical label sets within a single batch or even across the entire dataset.

To address this issue, we use an overlapping labels metrics. Particularly, to measure the degree of labels overlap between two sequences, we use the following metrics: 


(3) \begin{align*}& overlap_{ij} = \frac{\sum_{n=1}^{|L|} \min(y_{in}, y_{jn})}{\sum_{n=1}^{|L|} \max(y_{in}, y_{jn})}\end{align*}

Where $Overlap_{ij}$ denotes the similarity of labels in sequence i and sequence j. $|L|$ denotes the size of the considered label space and $y_{in}$ is the label of the sequence i for the class n.

With this concept of overlapping labels, we use an threshold of overlap, above which we consider two sequences as similar; otherwise, they belong to the distinct sequences category during contrastive training. Supplementary Section 1 describes a detailed example of the concept of overlapping labels.

Thus, with Algorithm 4, we handle the multi-label supervised contrastive training.


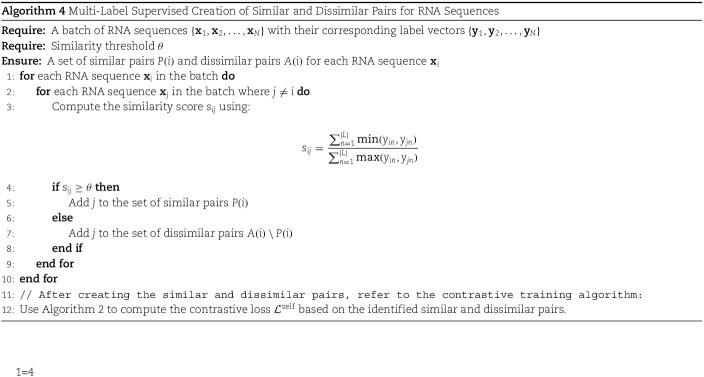


#### Prediction head: ML-decoder

For generating final predictions, we use the ML-Decoder [28] instead of standard classification layers. ML-Decoder is optimized for multi-label classification, making it more efficient and scalable for tasks where an input can belong to multiple classes. Unlike typical transformer decoders that use self-attention, ML-Decoder reduces computational demands by removing this mechanism, dropping the cost from quadratic to linear in the number of classes [28]. The removal of self-attention in ML-Decoder is based on the observation that during inference, the self-attention module of a transformer decoder provides a fixed transformation on the input queries, making it redundant. This redundancy arises because, as the queries enter the cross-attention module, they first pass through a projection layer before undergoing the attention operation. This projection layer is capable of transforming the queries into any desired representation, effectively rendering the self-attention mechanism unnecessary. By eliminating self-attention, ML-Decoder maintains the same expressivity as a standard transformer-based classification head without any degradation in performance. Furthermore, this removal significantly reduces the computational complexity of the model. Traditional transformer decoders have a quadratic dependency on the number of queries due to self-attention, making them computationally expensive, especially for tasks involving a large number of labels. By removing self-attention, ML-Decoder reduces this dependency from quadratic to linear, making it more scalable and efficient for multi-label classification tasks. The empirical validation provided in the original ML-Decoder study further confirms that this modification does not compromise classification accuracy while significantly improving inference efficiency. This efficiency gain is particularly beneficial in our task, where RNA sequences can belong to multiple localization classes, making ML-Decoder a practical choice over standard transformer decoders.

The ML-Decoder uses a cross-attention mechanism, where a set of group queries interacts with spatial embeddings produced by a backbone network. Here, the backbone network is derived from our encoder module, which processes the features of the RNA sequence. This encoder has been fine-tuned with a supervised contrastive loss to better capture relationships between labels. The encoder’s output becomes the input to the ML-Decoder.

In ML-Decoder, each group query taps into specific features from these embeddings, streamlining the model’s handling of multiple labels. The output of this interaction goes through a simple layer to yield the final class scores or “logits,” which indicate the likelihood of each class being present in the RNA sequence.

To train the ML-Decoder, we minimize binary cross-entropy loss, which encourages accurate predictions for each potential label in the input sequence. The SCL step helps the encoder understand label similarities, enhancing the model’s performance on complex, multi-label data.

### Complete training pipeline of LOCAS

**Figure 1:**
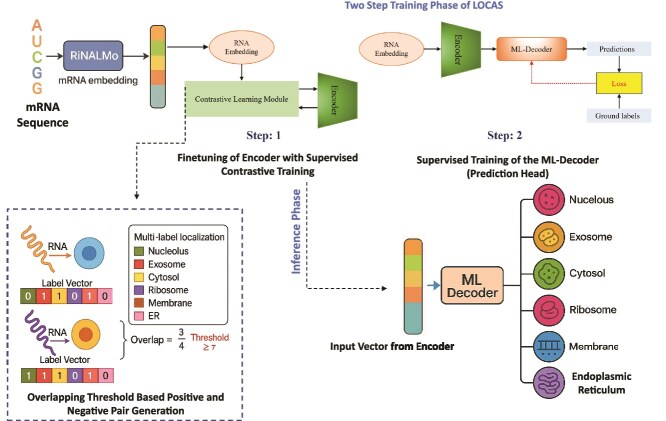
Overall training pipeline for LOCAS. In step 1, the encoder is finetuned with SCL. In the second step the encoder’s output is fed into the ML-decoder for final prediction.

With the necessary components introduced, the overall training pipeline of LOCAS consists of two main stages: SCL for encoder fine-tuning and supervised classification training of the ML-Decoder, as depicted in Fig. 1. These stages are designed to first refine the encoder’s feature representation using contrastive learning and then train the ML-Decoder for accurate multi-label classification.

**Step 1: Finetuning the Encoder with Supervised Contrastive Learning.** The training process begins with embedding generation using a pre-trained RNA language model (RiNALMo [27]). The RNA sequence is first transformed into a dense numerical representation using the language model. These embeddings serve as the feature representation for the RNA sequence. Once the initial embeddings are obtained, the encoder is trained using SCL as described in Section 2.1.3. The purpose of this step is to structure the latent space such that RNA sequences sharing localization labels are positioned closer together, while those with distinct localization labels are pushed apart. Given the multi-label nature of the problem, where an RNA sequence may belong to multiple localization sites, a similarity-based strategy is applied. Instead of treating all labels equally, an overlap-aware similarity score is used to determine positive and negative pairs. Sequences sharing localization labels above a predefined threshold are assigned as positive pairs, while others are considered negative pairs.

The encoder is optimized using a supervised contrastive loss function, which ensures that biologically relevant relationships are captured in the learned feature space. The optimization is performed using gradient-based methods, and the training continues until the representations exhibit well-separated clusters based on localization. After training, the encoder is frozen to preserve the learned feature structure for the next stage.

**Step 2: Supervised Training of the ML-Decoder.** Once the encoder is fine-tuned, it is used to extract embeddings for RNA sequences, but its weights remain frozen during this stage. The extracted embeddings are passed into the ML-Decoder, which serves as the classification head. Unlike traditional MLP-based classification layers, ML-Decoder leverages a cross-attention mechanism that dynamically learns label dependencies. This structure allows it to efficiently handle multi-label classification by learning to decode label-specific information from the extracted embeddings. The ML-Decoder processes the embeddings and produces logits corresponding to the likelihood of each RNA sequence belonging to different localization sites. These predicted logits are compared with the ground truth multi-label annotations, and a classification loss (binary cross entropy loss) is computed. The loss is then backpropagated through the ML-Decoder to refine its ability to classify RNA sequences accurately. Since the encoder remains frozen during this phase, only the classification head is updated.

After completing both training stages, the final model consists of the frozen fine-tuned encoder and the trained ML-Decoder. During inference, the initial embeddings for an RNA sequence is obtained from the RNA language model and then processed through the encoder to generate embeddings, which are then passed to the ML-Decoder for localization prediction. This pipeline ensures an effective combination of learned sequence representations and multi-label classification, making LOCAS a robust framework for RNA sub-cellular localization. Supplementary Section 4 depicts the detailed **Ablation Study** to validate our design choices of the methodology. Supplementary Section 5 contains the **hyperparameters of LOCAS** to facilitate reproducibility.

## Results

In this section, we present mRNA localization predictive performance on two benchmark datasets—RNALocate v1.0 and RNALocate v2.0. Next, we assess the predictive performance of LOCAS and state-of-the-art baseline models for each subcellular compartment individually, using MCC scores to benchmark localization accuracy across all target classes. Supplementary Section 2 contains the definition of the evaluation metrics. Moreover, to better understand the projection space of the SCL framework, we perform a clustering based on the output embedding from the encoder network, as presented in Supplementary Section 3.

### LOCAS outperforms state-of-the-art methods on both RNALocate v1.0 and RNALocate v2.0

**Figure 2:**
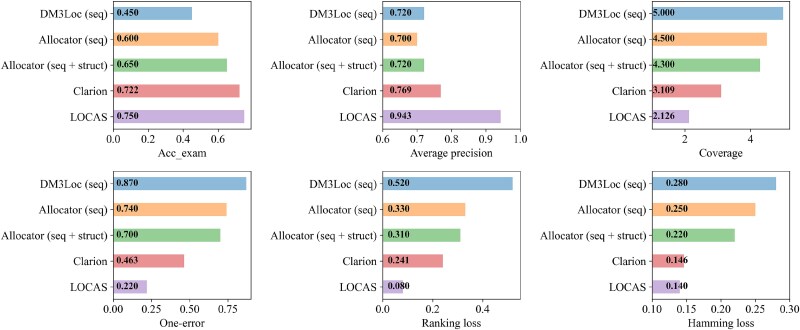
Performance comparison of LOCAS with other state-of-the-art methods for mRNA subcellular localization.

We first compare the performance of LOCAS on the RNALocate v1.0 dataset. For experiments in this dataset, we performed five-fold cross-validation similar to [5]. The comparative analysis of LOCAS with other state-of-the-art methods for RNA subcellular localization in Fig. 2—DM3Loc (seq), Allocator (seq), Allocator (seq + struct), and Clarion—reveals that LOCAS significantly outperforms these models across most evaluation metrics. In terms of accuracy, LOCAS achieves an example based accuracy (AccExam) of 0.75, which is the highest among the compared models. This improvement is notable compared to Clarion, which has the second-highest AccExam score of 0.722. The difference suggests that LOCAS is more effective in correctly identifying the subcellular localization of RNA sequences.

LOCAS also demonstrates superior performance in terms of average precision, achieving a score of 0.9434, which is a marked improvement over all other models. Clarion, the next best model, scores 0.769, while Allocator (seq + struct) achieves 0.72, highlighting that the precision of LOCAS in predicting the correct subcellular localization is significantly higher. This indicates that LOCAS is not only more accurate but also more reliable in consistently providing correct classifications across different samples.

In terms of coverage, LOCAS scores the lowest at 2.1262, compared to Clarion at 3.109 and Allocator models at 4.5 and 4.3. A lower coverage score reflects better performance, as it means that fewer incorrect or unnecessary predictions were made. This reduction in coverage error shows that LOCAS effectively minimizes the total number of predictions required to capture all relevant labels, suggesting that it achieves a better balance between precision and recall compared to other models.

LOCAS also significantly reduces the one-error rate to 0.0092, indicating that it rarely ranks an incorrect label higher than any correct labels. This is a substantial improvement over Clarion (0.463) and especially over the Allocator models (0.74 and 0.70) and DM3Loc (seq) (0.87). The low one-error rate of LOCAS demonstrates its strength in accurately ranking the most likely subcellular localizations at the top, thereby minimizing the risk of prioritizing incorrect localizations.

Additionally, LOCAS achieves the lowest ranking loss of 0.0804, far outperforming Clarion (0.241) and Allocator (seq + struct) (0.31). Ranking loss reflects how well a model ranks the true labels over the false ones, and the substantial reduction in ranking loss by LOCAS suggests that it is more adept at distinguishing between multiple possible labels and correctly ordering them.

Finally, the hamming loss for LOCAS is also the lowest at 0.1401, compared to Clarion at 0.146 and significantly lower than the Allocator models and DM3Loc (seq). This lower hamming loss indicates fewer incorrect classifications across all possible labels, further affirming the overall robustness of LOCAS in the multi-label classification setting.

In addition to the RNALocate v1.0, we have expanded LOCAS and trained it with the RNALocate v2.0 dataset, which comprises of a larger dataset covering more cellular locations. RNALocate v2.0 contains a separate independent test dataset that is used to benchmark to different methods. Table 2 presents a comparative evaluation of different localization prediction methods on RNALocate v2.0. The metrics used for evaluation include Aiming, Coverage, Accuracy, Absolute-true, and Absolute-false. Aiming (Precision) measures the average proportion of correctly predicted labels among all predicted labels for each sample, reflecting how precise the model is. Coverage (Recall) captures the average proportion of relevant (true) labels correctly predicted per sample, indicating the model’s ability to retrieve all true labels. Accuracy is computed using the Jaccard index, representing the average intersection-over-union between predicted and actual label sets. Absolute-True (Subset Accuracy) quantifies the percentage of samples for which the predicted label set exactly matches the ground truth. Finally, Absolute-False (Hamming Loss) measures the fraction of labels that are incorrectly predicted, providing insight into overall prediction errors across all samples and label dimensions. Among the evaluated methods, LOCAS demonstrates the best overall performance, achieving the highest Coverage (**0.715**), Accuracy (**0.577**), Absolute-true (**0.288**), and the lowest Absolute-false (**0.230**), making it the most reliable among the compared models. DM3Loc, while showing competitive Aiming (*0.7605*) and Accuracy (*0.4855*), has higher Absolute-false errors compared to LOCAS. Clarion performs best in Aiming (**0.7845**) and shows a relatively high Absolute-true score (*0.2823*). MSlocPRED excels in Coverage (*0.6780*) but falls short in other performance metrics.These results indicate that LOCAS provides a more balanced and accurate RNA localization prediction compared to the existing methods. Finally, through extensive evaluation on two benchmark datasets and comparison with state-of-the-art methods, LOCAS consistently achieves the best results, demonstrating its robustness and generalizability.

**Table 2:** Performance comparison of different methods on RNALocate v2.0

**Method**	**Aiming**	**Coverage**	**Accuracy**	**Absolute-true**	**Absolute-false**
DM3Loc	0.7605	0.5327	0.4855	0.2650	0.2338
Clarion	**0.7845**	0.4175	0.4168	0.2823	0.2683
MSlocPRED	0.6060	0.6780	0.4503	0.2093	0.3338
mRNALoc	0.7311	0.5146	0.3004	0.2178	0.2697
iLoc-mRNA	0.7095	0.5085	0.4762	0.2573	0.2567
RNATracker	0.7438	0.5229	0.4178	0.2457	0.2664
Allocator	0.7566	0.4353	0.4475	0.2612	0.2408
LOCAS	0.747	**0.715**	**0.577**	**0.288**	**0.230**

### LOCAS demonstrates strong class-wise localization accuracy

Table 3 presents a comparative analysis of various RNA localization prediction methods using Matthews Correlation Coefficient (MCC) across six subcellular compartments: Nucleus, Exosome, Cytosol, Ribosome, Membrane, and Endoplasmic Reticulum (ER) for the **RNALocate v1.0 dataset**. Each method’s performance is evaluated based on its MCC values, with the Avg MCC column representing the average across the available compartments.

**Table 3:** Comparison of MCC values across different methods on RNALocate v1.0 dataset

**Method**	**Nucleus**	**Exosome**	**Cytosol**	**Ribosome**	**Membrane**	**ER**	**Avg MCC**
DM3Loc	0.386	0.074	0.287	0.355	*0.312*	*0.205*	0.270
RNATracker	0.345	0.000	0.138	0.270	0.193	0.000	0.158
mRNALoc	0.150	0.000	−0.029	–	–	−0.148	−0.009
iLoc-mRNA	0.052	–	0.025	0.390	–	0.376	0.211
MSlocPRED	*0.3778*	*0.1700*	**0.6831**	*0.6145*	0.6388	**0.7205**	*0.5341*
**LOCAS**	**0.621**	**0.566**	*0.614*	**0.657**	**0.680**	*0.591*	**0.621**

Among the baseline methods, MSlocPRED performs relatively well, achieving the second-highest average MCC (*0.5341*). It excels in Cytosol (0.6831) and ER (0.7205), surpassing all other methods in these two compartments. However, its performance is inconsistent across other compartments, particularly in Exosome (*0.1700*) and Ribosome (*0.6145*), which prevents it from achieving state-of-the-art performance across all categories. DM3Loc, another competitive baseline, shows moderate performance with an average MCC of 0.270, performing decently in Nucleus (0.386) and Ribosome (0.355), but struggling in Exosome (0.074) and ER (0.205). RNATracker exhibits an overall weak performance, particularly failing in Exosome and ER (both MCC values are 0.000), resulting in a low average MCC of 0.158.

Other baseline methods, such as mRNALoc and iLoc-mRNA, show inconsistent and generally weaker performance. mRNALoc achieves an extremely low average MCC of -0.009, with negative performance in Cytosol (-0.029) and ER (-0.148), making it unsuitable for reliable RNA localization prediction. iLoc-mRNA, on the other hand, shows competitive performance in Ribosome (0.390) and ER (0.376), but due to missing predictions in Exosome and Membrane, its average MCC remains low at 0.211.

In contrast, our proposed method, LOCAS, establishes itself as the best-performing model, achieving the highest overall average MCC of 0.621. LOCAS significantly outperforms all baseline methods in Nucleus (0.621), Exosome (0.566), Ribosome (0.657), and Membrane (0.680), demonstrating its ability to provide accurate and consistent predictions across multiple compartments. Although MSlocPRED slightly surpasses LOCAS in Cytosol and ER, the differences are marginal (0.6831 vs. 0.614 for Cytosol and 0.7205 vs. 0.591 for ER). The overall performance of LOCAS remains superior due to its robustness across multiple compartments, unlike MSlocPRED, which has noticeable weaknesses in Exosome and Ribosome.

These findings confirm that LOCAS provides the most reliable RNA localization predictions, offering the best trade-off between accuracy and consistency across different subcellular compartments. While minor improvements can be explored in Cytosol and ER localization, the strong overall performance across all other compartments solidifies LOCAS as the new state-of-the-art approach for RNA localization prediction.

## Conclusion

In this work, we present LOCAS, a deep learning framework for accurate RNA subcellular localization prediction. By integrating a tailored RNA language model with SCL, LOCAS structures the feature space to form biologically meaningful clusters among RNAs with overlapping localization. This reduces misclassification due to label ambiguity and captures subtle inter-compartment relationships. The RNA language model further enriches contextual understanding beyond simple sequence similarity. Extensive ablation and benchmark evaluations demonstrate LOCAS’s superior performance, particularly for multi-compartment RNAs. Accurate multi-label localization is vital for understanding RNA function, regulation, and interaction—paving the way for insights into disease mechanisms, biomarker discovery, and RNA-based therapies.

Despite these advancements, our approach has certain limitations that warrant further exploration. First, the batchwise nature of supervised contrastive loss optimization may lead to inconsistencies in training effectiveness. Unlike unsupervised contrastive learning, which guarantees similar pairs in every batch, our model requires sequences from similar classes within each batch for optimal training. In cases where batches lack sufficient samples from the same class (as determined by the overlapping threshold), the contrastive learning component may underperform. Addressing this challenge could involve developing a batching strategy that ensures sufficient intra-class sequences in each batch, potentially improving the consistency of representation learning across training epochs.

Overall, LOCAS establishes a robust foundation for RNA localization prediction. We hope our contributions inspire further innovation in RNA-based localization research, paving the way for enhanced understanding of cellular processes and their implications in health and disease.

**Key Points**

- We present LOCAS, a novel deep learning framework, for mRNA sub-cellular localization prediction from RNA sequence. For mRNA sub-cellular localization task, we integrate the RNA language model for the first time to generate embedding space for the RNA sequences.
- We use an effective supervised contrastive learning (SCL) algorithm to identify the distinct clusters of the RNA sequences. As it is an multi-label classification task, overlap between the labels is natural, and to handle this overlap efficiently, we use an overlapping threshold based similarity score to identify the similar clusters during the supervised contrastive learning (SCL) training.
- We employ a specialized classification head designed for multi-label tasks (ML-Decoder), which uses a cross-attention mechanism while performing classification.
- We provide interpretation of the performance of LOCAS based on overlapping based similarity threshold.

## Supplementary Material

supplementary_bbaf441

## Data Availability

The datasets and the code are publicly available at: https://github.com/abrarrahmanabir/LOCAS.
