# Supplementary material for: LOCAS: multilabel mRNA localization with supervised contrastive learning
Source: Brief Bioinform. 2025 Aug 27;26(4):bbaf441. doi: 10.1093/bib/bbaf441 (PMC12381764; doi:10.1093/bib/bbaf441)
Supplement: supplementary_bbaf441 [file supplementary_bbaf441.pdf]

Supplementary Material for
LOCAS: Multilabel mRNA Localization with Supervised Contrastive Learning

Abrar Rahman Abir[†1], Md Toki Tahmid[†1], and M Saifur Rahman[*1]
[1]Department of Computer Science and Engineering,
Bangladesh University of Engineering and Technology, Dhaka, Bangladesh
[*]Corresponding author: mrahman@cse.buet.ac.bd
[†]Contributed equally

Supplementary Section 1. Example of Overlapping Labels

In subcellular localization task, where the possible labels are: Nucleolus, Exosome, Cytosol, Ribosome, Membrane, and Endoplasmic Reticulum (ER). Each RNA sequence can have a combination of these labels.

For example, consider the following label vector for an RNA sequence:

$$\text{Label Vector: } [0, 1, 1, 0, 1, 0]$$

This label vector indicates that the RNA sequence is localized in the Exosome, Cytosol, and Membrane.

There is a issue with this strict definition of class similarity. In a multi-label contrastive learning setting, the similarity between two RNA sequences is determined based on their label vectors. However, for the two RNA sequences to be considered as positives (i.e., similar pairs), their label vectors must be identical. For instance:

$$\text{RNA 1: } [0, 1, 1, 0, 1, 0]$$

$$\text{RNA 2: } [0, 1, 1, 0, 1, 0]$$

Here, RNA 1 and RNA 2 would be considered as positives since their label vectors are identical. However, consider another RNA sequence with the label vector:

$$\text{RNA 3: } [1, 1, 1, 0, 1, 0]$$

This vector indicates localization in the Nucleolus, Exosome, Cytosol, and Membrane. Although RNA 3 shares four out of the six labels with RNA 1, it would not be considered a positive pair with RNA 1 under the standard SupCon loss because their label vectors are not exactly the same. This strict requirement for identical label vectors can lead to a failure in recognizing partial similarities between RNA sequences, which reduces the generalization capability of the model and limits the number of positive pairs available for training.

For the given RNA 1 and RNA 3 labels above, the value of $Overlap_{13}$ will be as follows:

$$Numerator : min(0,1) + min(1,1) + min(1,1) + min(0,0) + min(1,1) + min(0,0)$$
$$Denominator : max(0,1) + max(1,1) + max(1,1) + max(0,0) + max(1,1) + max(0,0)$$

Thus, $Overlap_{ij} = \frac{3}{4}$ which is the degree of similarity between them.

Supplementary Section 2. Evaluation Metrics

In the context of RNA subcellular localization, each RNA sequence may be associated with multiple subcellular compartments, such as the nucleus, cytosol, or ribosome. Evaluating the performance of models that predict these locations requires specialized metrics tailored to multilabel classification. The following are key metrics used in this domain:

Example-Based Accuracy (AccExam) measures the accuracy per RNA sequence, considering all predicted subcellular localizations simultaneously. It is defined as the proportion of correctly predicted localizations out of the total possible localizations for each RNA sequence, averaged over all sequences:

$$\text{AccExam} = \frac{1}{N}\sum_{i=1}^{N}\frac{|Y_i \cap \hat{Y}_i|}{|Y_i \cup \hat{Y}_i|}$$

where Y_i is the set of true subcellular localizations, $\hat{Y}_i$ is the set of predicted localizations for the i-th RNA sequence, and N is the total number of sequences.

Average Precision evaluates the model's ability to rank relevant subcellular localizations higher than irrelevant ones. It is computed by averaging the precision obtained after each relevant localization is retrieved across all RNA sequences:

$$\text{Average Precision} = \frac{1}{N}\sum_{i=1}^{N}\frac{1}{|Y_i|}\sum_{k=1}^{|Y_i|}\text{Precision@k}(Y_i)$$

where Precision@k(Y_i) is the precision at the k-th relevant localization.

Coverage measures how far we need to go down the ranked list of localizations to cover all true subcellular locations for each RNA sequence. It is defined as:

$$\text{Coverage} = \frac{1}{N}\sum_{i=1}^{N}\max_{y \in Y_i}\text{rank}(y) - 1$$

where rank(y) is the rank of the true localization y in the predicted ranking.

One-Error indicates the proportion of RNA sequences for which the top-ranked subcellular localization is not among the true localizations. It reflects the model's ability to correctly identify the most likely localization:

$$\text{One-Error} = \frac{1}{N}\sum_{i=1}^{N}\mathbb{I}\left(\text{argmax}_y\, \hat{y}_i \notin Y_i\right)$$

where $\mathbb{I}(\cdot)$ is the indicator function that returns 1 if the top localization is incorrect, and 0 otherwise.

Ranking Loss calculates the average proportion of incorrectly ordered pairs of subcellular localizations, i.e., cases where an irrelevant localization is ranked higher than a relevant one:

$$\text{Ranking Loss} = \frac{1}{N}\sum_{i=1}^{N}\frac{1}{|Y_i||\overline{Y_i}|}\sum_{y \in Y_i}\sum_{y' \in \overline{Y_i}}\mathbb{I}(\text{rank}(y) > \text{rank}(y'))$$

where $\overline{Y_i}$ is the set of irrelevant localizations for the i-th RNA sequence.

Hamming Loss is the fraction of subcellular localizations that are incorrectly predicted. It counts both false positives (irrelevant localizations predicted as relevant) and false negatives (relevant localizations not predicted):

$$\text{Hamming Loss} = \frac{1}{N}\sum_{i=1}^{N}\frac{1}{L}\sum_{j=1}^{L}\mathbb{I}(y_{ij} \neq \hat{y}_{ij})$$

where L is the total number of possible subcellular localizations, and y_{ij} and $\hat{y}_{ij}$ are the true and predicted binary labels for the i-th RNA sequence and the j-th localization, respectively.

Supplementary Section 3. LOCAS Embedding Space Reflects Label-Aware Clustering

To better understand the projection space of the supervised contrastive learning framework, we perform a clustering based on the output embedding from the encoder network. Unsupervised projection helps to understand if the overlapping similarity based contrastive loss is reflected in the encoder projection. To understand the clustering difference of the positive and negative samples from each class, we project the output of the encoder to two dimension using t-SNE and create a 2D projection visualization as shown in Figure 1. In Figure 1, we see the distribution of the positive samples from each different class. Among the six different classes, exosome has the most number of positive count, and encoplasmic reticulum consists of the least number of positive sample. Nucleus, cytosol and ribosome has an overall equal distribution of positive and negative samples. This distribution pattern actually impacts the supervised contrastive loss. We see in Figure 1 that, for nucleus, cytosol, and ribosome the clustering from the t-SNE plot provides a clear distinction. However, for the endoplasmic reticulum class, the encoder network struggles to capture the separation between the positive and the negative class.
black These clustering patterns provide insight into how well the supervised contrastive learning framework has structured the feature space. The clear separation observed in nucleus, cytosol, and ribosome suggests that the encoder successfully captures discriminative features for these classes, which improves classification performance as shown in Table 1. In contrast, the poor separation in the endoplasmic reticulum class indicates that the learned representation struggles to distinguish its samples, potentially due to overlapping features with other localization sites. The impact of class imbalance is also evident in exosome and endoplasmic reticulum, where the smaller number of positive samples results in weaker clustering. This suggests that the supervised contrastive loss is more effective when the number of positive and negative samples is relatively balanced. The varying degrees of separation across classes highlight the challenges of RNA localization prediction and emphasize the importance of incorporating label similarity information into the training process. The t-SNE visualization thus provides qualitative validation that the encoder's learned representation.

3.1 LOCAS Captures Semantic Label Proximity with Overlap Thresholding

We actually do not consider strictly the labels as they are distinctly represented in the dataset. Rather we consider an overlapping threshold with which we decide whether two different sequences belong to the same class even though their labels do not exactly match. With this approach,we can construct an adjacency matrix using the following formulation:

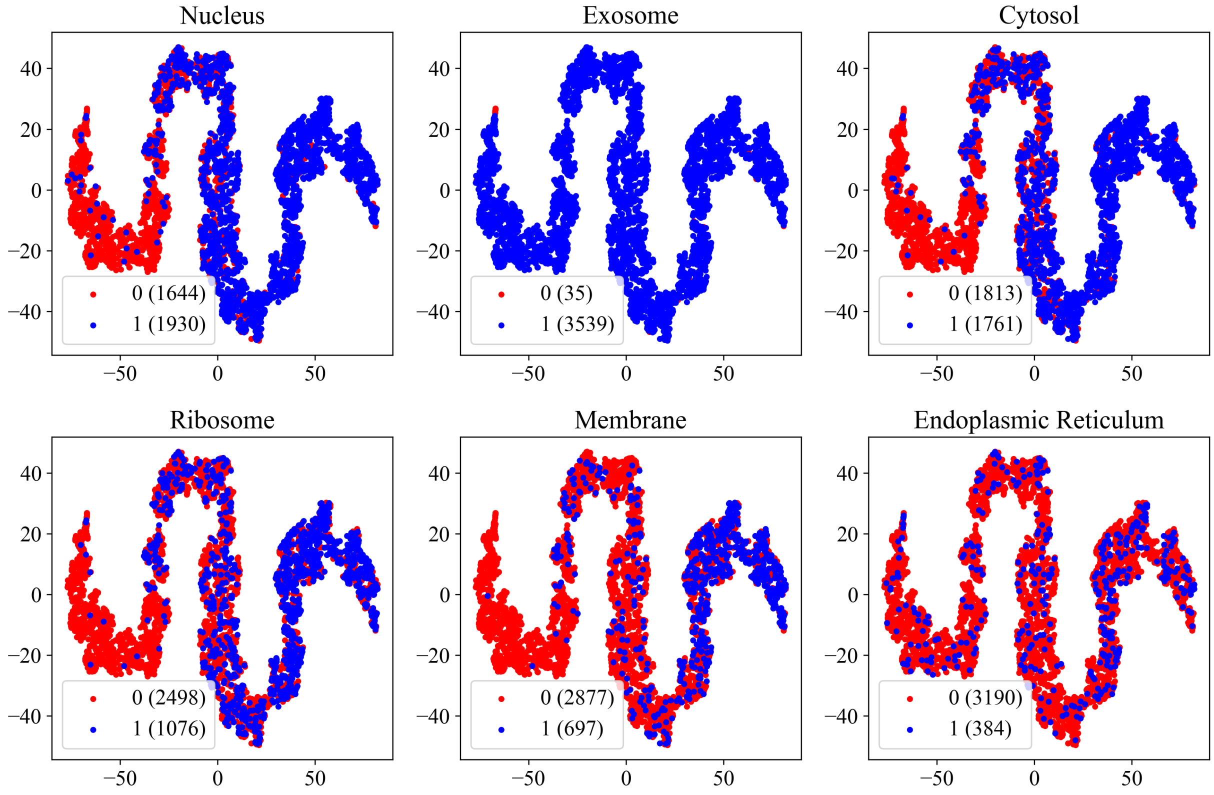

Figure 1: t-SNE visualization for each Class (0 vs 1). The number of positive and negative samples in each class is also shown in the legend.

Let S_i and S_j be two sequences with their corresponding label sets L_i and L_j. Define the overlap function $\text{Overlap}(L_i, L_j)$ as the ratio of the intersection to the union of these label sets:

$$\text{Overlap}(L_i, L_j) = \frac{|L_i \cap L_j|}{|L_i \cup L_j|}$$

Now, define the adjacency matrix A where each element A_{ij} is determined by whether the overlap between the label sets of two sequences exceeds a predefined threshold τ:

$$A_{ij} = \begin{cases} 1, & \text{if Overlap}(L_i, L_j) \geq \tau \\ 0, & \text{otherwise} \end{cases}$$

Using this adjacency matrix A, we can compute the connected components, which represent the number of unique classes formed based on the overlapping threshold τ. We try to understand, if this overlapping based classification is reflected in the output of the encoder network. blackWe see in Figure 3 that varying the overlapping threshold significantly alters the number of connected components, which corresponds to changes in the number of distinct classes formed. A lower threshold results in more isolated clusters, preserving strict class boundaries, whereas a higher threshold merges sequences into broader groups, capturing shared localization features. This threshold-based clustering mechanism provides a more flexible and biologically relevant way to define RNA localization groups by accounting for functional similarities that might not be explicitly captured in the dataset's predefined labels. The variation in the number of connected components directly impacts

the contrastive learning process, as it influences how the encoder structures the feature space and forms meaningful clusters for multi-label classification. As discussed in Section 4.2, these threshold-driven changes affect both the supervised contrastive loss and the final classification performance. The number of connected components determines how contrastive pairs are selected, which in turn influences the encoder's ability to learn discriminative features. A lower threshold enforces a stricter contrastive objective, leading to better-defined clusters but potentially overlooking shared localization properties. In contrast, a higher threshold increases the number of positive pairs in the contrastive loss, promoting more generalizable feature representations but potentially reducing class separability. Despite these threshold-based modifications, during downstream fine-tuning for the actual classification task, model evaluation follows a class-based binary classification approach, where each RNA sequence is independently classified into one of six localization classes: nucleus, exosome, cytosol, ribosome, membrane, or endoplasmic reticulum. Understanding the influence of threshold variation is essential, as it highlights the trade-off between fine-grained and generalized classification decisions, ultimately shaping the model's predictive performance.

Supplementary Section 4. Ablation Study

To understand the superior performance of LOCAS over the previously proposed methods and to investigate the impacts of different modules implemented in the architecture, we perform a comprehensive ablation study. First, we look into the impact of the contrastive learning approach on the overall downstream performance of LOCAS. Moreover, we analyze the importance of choosing the right overlapping threshold with both clustering space understanding and performance comparison.

4.1 Contrastive Learning Drives Major Gains in LOCAS

In Figure 2, we have shown the impact of the supervised multilabel contsrative loss. We see that, without the SCL, we obtain an inferior performance over all the metrics. It is to be noted that, in this figure we have reported the mean score over all class types. In Table 2, we show the class-wise performance analysis of with and without SCL. We see that a significant degradation in performance (MCC score) is observed when we do not use the SCL (Supervised Contrastive Learning)approach. Without SCL, we get an MCC score of zero for three classes. Moreover, for nucleus, we get an MCC score of 0.142 with LOCAS which is inferior to most of the methods reported in Table 1. The same trend is observed in the case of the ribosome and cytosol, also indicating a great importance of learning the hidden contextual information of RNAs with a label-informed multi-label contrastive loss.

Table 1: Comparison of MCC values across different methods on RNALocate v1.0 dataset.

**Method**	**Nucleus**	**Exosome**	**Cytosol**	**Ribosome**	**Membrane**	**ER**	**Avg MCC**
DM3Loc	0.386	0.074	0.287	0.355	*0.312*	*0.205*	0.270
RNATracker	0.345	0.000	0.138	0.270	0.193	0.000	0.158
mRNALoc	0.150	0.000	-0.029	-	-	-0.148	-0.009
iLoc-mRNA	0.052	-	0.025	0.390	-	0.376	0.211
MSlocPRED	*0.3778*	*0.1700*	**0.6831**	*0.6145*	0.6388	**0.7205**	*0.5341*
**LOCAS**	**0.621**	**0.566**	*0.614*	**0.657**	**0.680**	*0.591*	**0.621**

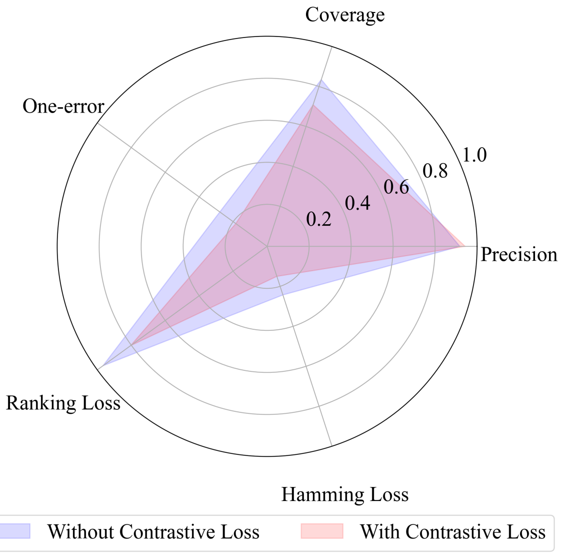

Figure 2: Difference in performance with and without using the supervised contrastive loss (across all classes).

**Class**	**Without SCL**	**With SCL**
Nucleus	0.142	0.621
Exosome	0.000	0.566
Cytosol	0.130	0.614
Ribosome	0.005	0.657
Membrane	0.000	0.591
End. Reticulum	0.000	0.621
**Average**	**0.046**	**0.433**

Table 2: MCC values for each class with and without contrastive loss.

4.2 Overlap Threshold Controls Cluster Diversity and Performance

As we show in Figure 3, for different threshold values we obtain varied number of distinct clusters. In Table 3, we show the number of connected components (distinct labels) and the clustering performance for different contrastive learning thresholds (θ). We also show the distribution of number of RNA sequences in each cluster. For θ= 0.3, we get 13 different labels (clusters), however the distribution is highly biased towards only one single cluster and the other clusters contain just a single data point. For $\theta = 0.5$, we get 18 components with a similar type of biased distribution towards only one single class. However, for θ= 0.8, we see that there are 46 different clusters and the distribution also follows a long tailed distribution. We report the downstream performances for these different thresholds in Table 4. We see that, for $\theta = 0.8$, for which we get a better distribution of components, also provides the best performance.

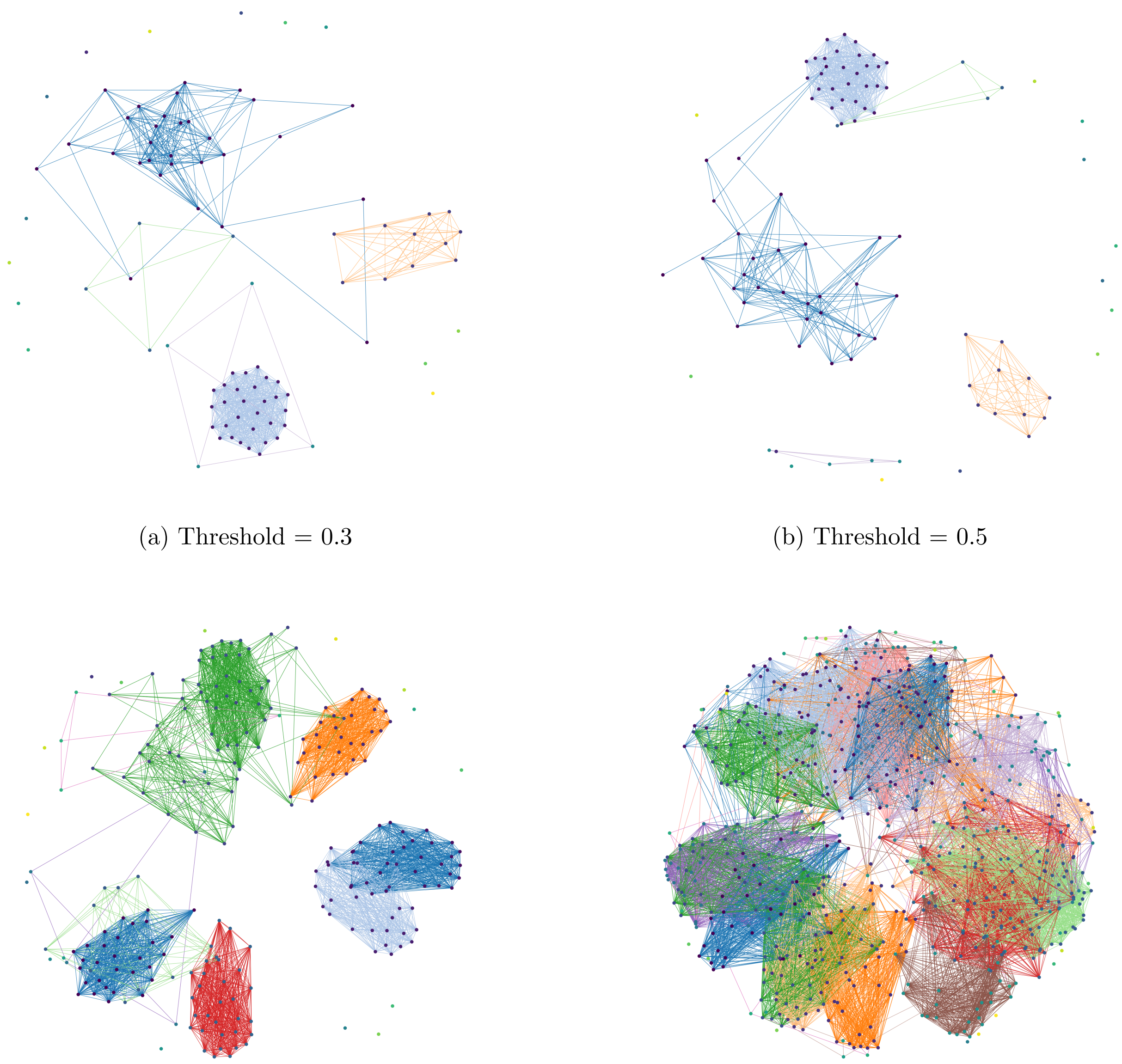

(a) Threshold = 0.3

(b) Threshold = 0.5

(c) Threshold = 0.6

(d) Threshold = 0.8

Figure 3: Changes in the number of distinct classes (number of connected components) with varying threshold value for labels overlap.

Supplementary Section 5. Hyperparameter Settings for Encoder and ML-Decoder

θ = **0.3**	θ = **0.5**	θ = **0.8**
Components = 13	Components = 18	Components = 46
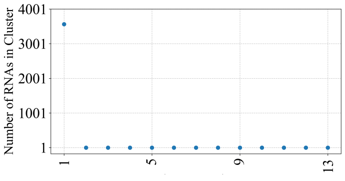	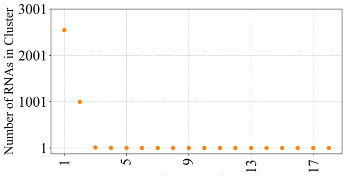	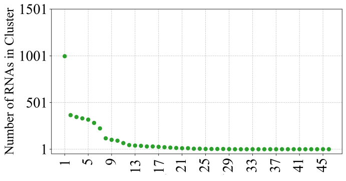
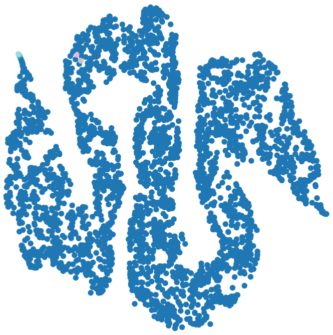	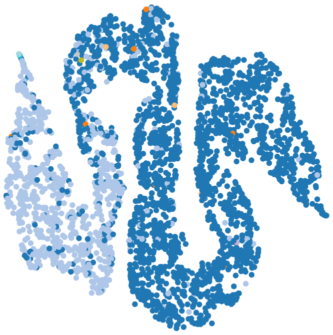	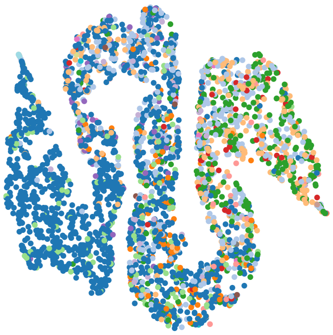

Table 3: Unsupervised Clustering with Different Overlapping Threshold.

Table 4: Performance Metrics for Different Values of θ

θ	**Average Precision**	**Coverage**	**One-error**	**Ranking Loss**	**Hamming Loss**
0.3	0.503	4.954	0.583	0.701	0.431
0.5	0.907	3.156	0.011	0.090	0.237
0.8	0.943	2.126	0.009	0.080	0.150

Table 5: Parameter settings for the Encoder and ML-Decoder

**Encoder Parameters**	
Component	Parameter
ResidualConvBlock-1	Input: 1, Output: 64, Kernel: 3
ResidualConvBlock-2	Input: 64, Output: 128, Kernel: 3
ResidualConvBlock-3	Input: 128, Output: 256, Kernel: 3
Multihead Attention-1	Embed Dim: 256, Heads: 8
Multihead Attention-2	Embed Dim: 256, Heads: 8
Fully Connected (Encoder Output)	Input: 256, Output: 128
**ML-Decoder Parameters**	
Component	Parameter
Transformer Decoder Layer	Embed Dim: 1280, Heads: 8
ML-Decoder Embedding	Dim: 1280
ML-Decoder Query Embedding	Queries: 100
ML-Decoder Feedforward Dim	Feedforward: 2048
ML-Decoder Num Layers	Layers: 1
ML-Decoder Dropout	Dropout: 0.1
